# Efficacy of a Waist-Mounted Sensor in Predicting Prospective Falls Among Older People Residing in Community Dwellings: A Prospective Cohort Study †

**DOI:** 10.3390/s25082516

**Published:** 2025-04-16

**Authors:** Ka-Ming Lai, Kenneth N. K. Fong

**Affiliations:** 1 Hong Kong Red Cross, Hong Kong SAR, China; martin.lai@redcross.org.hk; 2 Department of Rehabilitation Sciences, The Hong Kong Polytechnic University, Hong Kong SAR, China; 3 Research Centre for Assistive Technology, The Hong Kong Polytechnic University, Hong Kong SAR, China

**Keywords:** wearable sensors, fall risk prediction, older people, gerontechnology, fall prevention, digital health solutions, community-dwelling elderly

## Abstract

Falls pose a significant health risk for older people, necessitating accurate predictive tools for fall prevention. This study evaluated the sensitivity of a wearable waist-belt sensor, the Booguu Aspire system, in predicting prospective fall incidents among 37 community-dwelling older people in Hong Kong. A prospective cohort design was employed, involving two analytical groups: the overall cohort and a subset with cognitive performance data available, measured using the Montreal Cognitive Assessment (MoCA). Participants were categorized into moderate- or high-risk groups for falls using the sensor and further assessed with physical function tests, including the Single Leg Stand Test (SLST), 6 Meter Walk Test (6MWT), and Five Times Sit to Stand Test (5STS). Fall incidents were monitored for 12 months through quarterly follow-up phone calls. Statistical analyses showed no significant differences in physical performance between high- and moderate-risk groups and no significant correlations between sensor-based fall risk ratings and physical function test outcomes. The SLST, 6MWT, 5STS, and MoCA tests classified sensor-determined fall risk ratings with accuracies of 51.4%, 64.9%, 59.5%, and 50%. The sensor showed low sensitivity, with 13.51% true positives for fallers and a 20% sensitivity for high-risk individuals. ROC analysis yielded an Area Under the Curve of 0.688. Our findings indicate that the wearable waist-belt Sensor System may not be a sensitive tool in predicting prospective fall incidents. The algorithm for fall risk classification in the wearable sensor merits further exploration.

## 1. Introduction

Falls and fall-related injuries are major threats to the health and well-being of older people, and they incur heavy burdens on individuals, families, communities, and healthcare systems worldwide [1]. The global prevalence of falls among community-dwelling older people aged 65 years or older is estimated at 28–35% annually [2]. In Hong Kong, approximately 17% of older people experience falls each year, with 4% reporting recurrent falls. These incidents lead to significant physical injuries, psychological effects such as fear of falling, and loss of independence [3]. Falls frequently result in serious consequences, such as fractures, traumatic brain injuries, and premature mortality [4,5]. In addition to physical harm, falls can lead to a fear of falling that exacerbates anxiety and social isolation, further compromising quality of life and increasing caregiver burden [6,7]. In Hong Kong, fall-related public hospitalization for older people results in costs approximately HKD 552 million higher annually compared to non-fallers, due to increased numbers of visits to general outpatient departments, specialist clinics, accident and emergency departments, and longer hospital stays [8]. Moreover, fallers face a threefold higher 1-year institutionalization rate, highlighting long-term societal impacts [8]. However, recent evidence suggests that effective fall prevention programs could reduce falls and associated costs by up to 30%, underscoring the imperative for precise early detection and intervention [9].

Clinical judgment, balance tests, and self-reported questionnaires are traditional approaches to identify fall risk in apparently healthy seniors [10,11]. Examples of common assessments include the Timed Up and Go (TUG) test, Berg Balance Scale (BBS), and Falls Efficacy Scale (FES). These have been widely used and validated but often require a clinical setting and trained personnel to carry them out. They can also be influenced by subjective biases and environmental factors [11]. Subjective biases can stem from the clinician’s personal judgment and experience, which can vary widely between practitioners. This variability can lead to inconsistent assessments of fall risk, reducing the reliability of assessment results [12]. Moreover, various clinical assessments often measure different physical parameters, which may not provide a comprehensive assessment of fall risk. For example, the TUG primarily measures mobility and dynamic balance, the BBS focuses on static balance, and the FES evaluates psychological factors related to falls [13]. This fragmentation means that no single test can capture all dimensions of fall risk, potentially leading to incomplete fall risk assessment [11].

Recent advances in wearable sensor technology have facilitated objective, real-time monitoring of fall risk factors. Wearable technologies provide an opportunity to enhance fall risk assessment through continuous, objective, and real-life mobility monitoring [14,15]. Common wearables used in health monitoring include wristbands, smartwatches, and waist-belt sensors [16]. Waist-belt sensors are specifically designed for more targeted applications such as posture and balance monitoring as well as walking speed analysis. These devices contain accelerometers and gyroscopes that measure kinematics related to postural control and gait performance to inform detailed data on body movements and balance [17], which provides valuable data on mobility function and balance, for early identification of fall risks [18]. However, there is limited evidence regarding the predictive validity and clinical utility in forecasting prospective fall occurrences compared to the traditional method [19,20]. To be considered a viable alternative or complement to existing assessments, wearable technologies must demonstrate clear advantages in predictive accuracy, usability, and clinical feasibility [21].

The Booguu Aspire^TM^ Fall Risk Management System (the Sensor System) (Equipment: Booguu Company Limited (https://www.booguu.bio/aspire?lang=en, accessed on 22 March 2025), Hong Kong SAR, China), a commercial waist-belt wearable using inertial measurement unit in the market, was selected for this study primarily due to its user-friendly design, which allows for easy wearing by older people without the need for professional assistance. The device was one of the few waist-belt-like wearables available at the time in the market, which claimed to be able to identify fall risk, and with reports available to the local community and accessible by the customers. Users are required to engage in a series of standardized assessments while wearing the device, ensuring consistent data collection. A systematic review of the inertial sensors to evaluate geriatric fall risk found that inertial sensors were placed on the lower back in majority of the studies and variables such as position, angle, angular velocity, liner acceleration, energy, spatial and temporal, as well as frequency data have been used in predicting prospective falls, retrospective fall history and clinical fall assessments, however, the most promising sensor sites in identifying fallers was unclear [14]. A recent review on the use of wearable inertial sensors for fall risk assessment prediction in older adults found that the most effective feature to assess the risk of falling was the velocity with the inertial sensor placed on the shins during walking, or the linear acceleration measured at the lower back during standing or sit-to-stand and/or the stand-to-sit transfers [22]. Therefore, this study aimed to evaluate the Sensor System’s sensitivity and clinical utility in predicting fall risk over a prospective 12-month period, a gold standard of fall risk, among older people in Hong Kong. This study provided insights into the device’s practical applications and potential limitations, contributing to the broader understanding of similar wearables in the market for fall prevention, and the broader field of study in both research and clinical applications in the field of digital health for the ageing population.

## 2. Materials and Methods

### 2.1. Participants

A total of 37 community-dwelling older people in Hong Kong voluntarily participated in this study, recruited through convenience sampling during a fall risk screening program organized by the Hong Kong Red Cross in 2021. Participants were included if they met the following criteria: (1) age ≥ 60 years, as this age group is at higher risk for falls due to physiological changes and functional decline. (2) An ability to ambulate independently for at least 10 m with or without walking aids to ensure they could complete the physical performance tests. (3) An ability to communicate effectively in Cantonese or English, ensuring proper understanding of study procedures and instructions. Participants residing in residential care settings were excluded to maintain a homogeneous community-dwelling sample, as institutionalized older people often have different fall risk factors, levels of supervision, and mobility patterns compared to those living independently.

The sample consisted of 78.4% females and 21.6% males. Participants varied in occupation and activity level, with most being retired or engaged in low-intensity physical activities. The gender distribution aligns with epidemiological data indicating a higher prevalence of fall-related concerns among older women due to age-related muscle loss and balance impairments. A recent systematic review highlights that older females have a significantly higher fall risk and incidence than males, reinforcing the observed pattern in our sample [23]. However, subgroup analyses based on gender and occupation were not conducted due to the small sample size, limiting the ability to assess their specific impact on fall risk. 

Ethical approval was obtained from the Institutional Review Board of The Hong Kong Polytechnic University (Approval Code: HSEARS20230803001). All participants provided written and oral informed consent for study participation before enrollment.

### 2.2. Equipment: Sensor System

**Figure 1:**
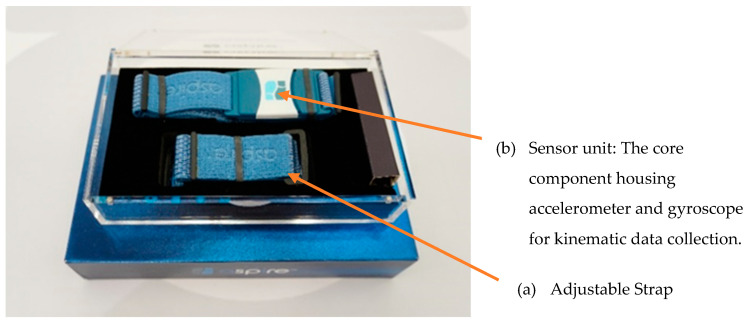
The waist-mounted wearable Sensor System.

**Figure 2:**
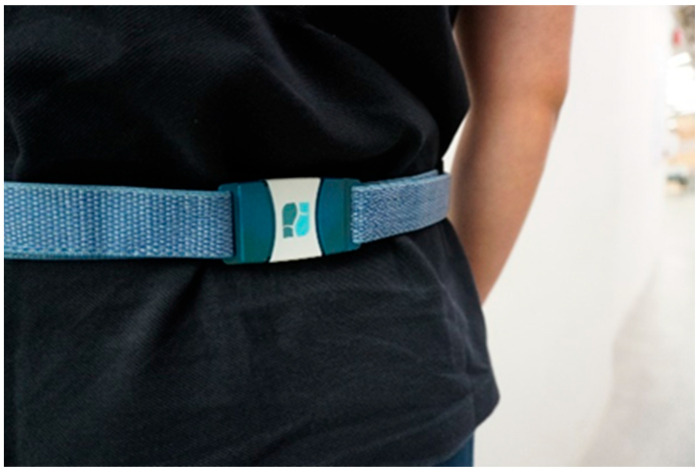
Positioning of the Sensor at the L5 vertebral level on the waist.

The Sensor System is a waist-belt-like wearable developed to provide feedback information regarding mobility and balance to the individual. It is 4 cm × 5.5 cm × 1.2 cm, with built-in accelerometers and gyroscopes (Figure 1 and Figure 2), and must be fastened with an elastic waist belt in a position over the L5 vertebra (lower back). This position ensures unobtrusive and precise data collection, capturing the intricate dynamics of posture and movement with a high sampling rate of 100 Hz [24]. The Sensor System utilizes a proprietary algorithm to evaluate key fall risk parameters, including balance, gait characteristics, and walking speed [17]. The device records kinematic movement data while participants perform standardized functional assessments. These data points are transmitted via Bluetooth to a tablet-based interface, where real-time analysis generates fall risk classification reports [17]. The output of the system categorizes participants into moderate- or high-risk groups based on predefined thresholds. However, the algorithm’s underlying methodology remains proprietary and was not disclosed by the manufacturer, limiting the ability to independently validate its computational approach. A recent study investigated the use of the Sensor in fall risk assessment of older adults. The results found that the Sensor has good test-retest reliability and internal consistency; however, it might not be sensitive to identify non-fallers and recurrent fallers retrospectively [17]. Therefore, the Sensor’s clinical effects in predicting prospective fall occurrences remain uncertain. This study aimed to assess its predictive validity by correlating sensor-classified risk levels with observed prospective fall incidents over 12 months.

### 2.3. Study Design and Data Acquisition

This study employed a prospective cohort design. Data acquisition utilized the Sensor System, with the sensor positioned at the L5 vertebra to collect high-resolution kinematic data on mobility and balance via integrated accelerometers and gyroscopes. Participants performed standardized physical tasks, including (i) maintaining balance with both eyes open and closed for 30 s to assess postural control, (ii) completing a 10 m walk at a comfortable pace to evaluate gait performance, and (iii) executing five sit-to-stand repetitions within 15 s to assess lower limb strength and functional mobility. Data collected from the sensor was transmitted in real-time via Bluetooth to a tablet-based interface, where the system’s fall risk assessment algorithm generated classification reports. However, the specific computational methodology behind the classification remains undisclosed by the manufacturer, limiting independent validation. To complement sensor-based assessments, participants who expressed cognitive concerns were invited to complete cognitive screening using the Hong Kong version of the Montreal Cognitive Assessment (MoCA) [25].

In addition to the sensor-based data, a set of physical performance tests was conducted to provide a multidimensional evaluation of fall risk. The Single Leg Stand Test (SLST) [26] was used to assess static balance and postural stability by measuring the duration a participant could stand on one leg without support. The 6 Meter Walk Test (6MWT) [27] was conducted to evaluate gait speed and endurance, requiring participants to walk at their usual pace over a marked six-meter distance. The Five Times Sit to Stand Test (5STS) [28] measured lower limb strength and mobility by instructing participants to rise from a chair five times as quickly as possible without using their arms for support. Participants were followed for 12 months prospectively through quarterly phone calls conducted by nursing staff. During follow-ups, they were asked about any fall incidents, the date of occurrence, and any associated injuries. This standardized fall monitoring method aligns with established research practices [10], ensuring reliable tracking of real-world fall occurrences.

### 2.4. Outcome Measures

The primary outcome was the occurrence of a fall during the 12-month follow-up period, as reported by participants through quarterly phone calls. Falls were defined as any event resulting in an unintentional change in position that led to the participant coming to rest on the ground or a lower surface. Secondary outcomes included the sensor’s sensitivity, specificity, and false positive rate in predicting falls, as well as its agreement with physical performance tests using the SLST, the 6MWT, and the 5STS. The number of medications used by the participants was recorded in the baseline because polymedication is one of the risk factors for falls [9].

### 2.5. Analytical Groups

Statistical analysis was divided into two groups based on the availability of cognitive performance data. Group 1 (n = 37) covered the entire cohort and aimed to correlate the sensor’s risk classifications with documented fall incidents over the study period. Group 2 (n = 22) focused on participants who underwent cognitive screening using MoCA; among them, 19 participants scored above the MoCA cut-off score of 21, indicating no significant cognitive impairment, while 3 participants scored below this threshold, suggesting potential cognitive decline [29]. Given the influence of cognitive function on fall risk, this subgroup analysis was conducted to explore whether cognitive status affected the sensor’s fall risk classification. The Sensor system stratified participants into three fall risk levels: low, moderate, and high risk. However, for this study, only individuals classified as moderate or high risk were included in the analysis, as they were considered to carry a higher fall risk warranting closer follow-up. Among Group 1, 67.6% of participants were classified as high risk, while 32.4% were categorized as moderate risk. In Group 2, a slightly higher proportion of 72.7% were classified as high risk, while 27.3% were categorized as moderate risk. This distribution suggested a potential trend of higher fall risk classification among individuals with cognitive concerns. Table 1 presents the demographic and baseline characteristics of all participants, providing context for the observed trends.

### 2.6. Data Analysis

Statistical software SPSS version 26 was used in the analysis. The relationships between the fall risk rating recorded by the sensors, performance on physical tests, and incidence of falls were investigated using Spearman’s correlation. This non-parametric test was chosen due to its suitability for detecting associations between ranked variables, particularly given the ordinal nature of the sensor’s risk classification. To explore differences in physical test performance between the high-risk and moderate-risk groups, independent t-tests were performed. Cohen’s d was calculated to quantify the effect size of these differences, allowing for an interpretation of the magnitude of differences in physical function between risk groups. Discriminant functional analysis evaluated the discriminant validity of physical performance tests and cognitive screening tools in differentiating fall risk levels. This approach helped determine whether these assessments could effectively classify individuals into sensor-determined risk categories. Logistic regression models were applied to examine the predictive ability of independent variables and the sensor’s fall risk classification for prospective falls within the 12-month period. Additionally, classification tables were generated to assess how well the sensor’s fall risk ratings corresponded with observed fall incidents. This analysis aimed to determine whether the sensor’s fall risk classification effectively predicted real-world fall occurrences. The sensitivity of the sensor in identifying prospective falls was analyzed based on the contingency table and the participants’ groups (high risk and moderate risk). The sensitivity of the sensor for identifying repeated falls within the high-risk group was analyzed based on the proportion of older people who experienced repeated falls to the total number of older people classified as high risk. Receiver Operating Characteristic (ROC) analysis was used to evaluate the overall accuracy of the sensor in distinguishing between fallers and non-fallers, with the Area Under the Curve (AUC) value used as a measure of the sensor’s classification performance, with higher values indicating greater predictive accuracy.

## 3. Results

### 3.1. Analysis of Physical Performance Differences by Risk Group Across Two Groups

The comparative analysis presented in Table 2 highlights the differences in the physical function test outcomes between the high-risk and moderate-risk groups across the two groups. In Group 1 (n = 37), the SLST indicated a slight mean difference between the high-risk (n = 9) and moderate-risk (n = 28) groups, with high-risk participants exhibiting shorter standing durations, indicative of poorer balance control. However, these differences did not reach statistical significance. The 6MWT and 5STS in Group 2 (n = 22) demonstrated more considerable mean differences, with the 6MWT showing slower walking speeds for the high-risk group (n = 5), aligning with an increased risk of falls. To elaborate on these differences, Table 2 provides a statistical examination of the physical performance between the two risk groups. In Group 1, the analysis revealed no significant differences between the two groups across all assessments. Similarly, for Group 2, there were no significant differences across all assessments between the two groups.

### 3.2. Discriminant Validity of Physical Performance Tests in Fall Risk Classification

By using the scores of SLST, 6MWT, and 5STS for predicting the classification of fall risk levels as determined by the wearable sensor, the results of discriminant validity indicated that 51.4%, 64.9%, and 59.5%, respectively, were correctly classified (Table 2).

### 3.3. Discriminant Validity of Cognitive Screening Tool in Fall Risk Classification

A separate table was created to present the discriminant validity of the cognitive screening tool, determined through discriminant functional analysis for Group 2. By using the MoCA to predict fall risk levels classification as determined by the wearable sensor, the results indicated that 50.0% of fall risk levels in Group 2 were correctly classified (Table 3).

### 3.4. Correlation Analysis of Physical Tests and Sensor Risk Classification

The results from Table 4 show the correlations between the SLST, 6MWT, 5STS, and the wearable sensor’s risk classification across two groups. In Group 1, correlations between these physical tests and the sensor’s risk classification did not reach statistical significance. Similarly, in Group 2, the correlations for the SLST, 6MWT, and 5STS continued to show no significant associations with the sensor’s risk classification.

### 3.5. Correlation Analysis and Logistic Regression Analysis of Fall Risk Predictors

Pearson’s correlation evaluates the correlation between the physical performance tests and the incidence of falls over the course of one year in each group. The results did not show any significant correlations for the SLST, 6MWT, or 5STS with the number of fall occurrences in either group. In addition, Logistic regression analysis did not reveal any statistically significant relationship between the physical performance tests and high fall risk classification by the sensor in terms of predicting those who would fall over the course of one year in either group.

### 3.6. Sensor Sensitivity

To determine the Sensor’s sensitivity for predicting prospective falls, we calculated several key statistical metrics and constructed a 2 × 2 contingency table to clarify these measures, as illustrated in Table 4. The predictive validity assessment showed varied outcomes based on the Sensor’s risk classification against actual fall events. Among participants classified as high risk, five individuals experienced falls (true positives), whereas 20 individuals did not fall (false positives). This resulted in a fall prediction rate of 20% among those classified as high risk. Conversely, in the moderate-risk group, none of the participants experienced a fall, leading to a fall prediction rate of 0% for this group. The absence of unexpected falls within the moderate-risk category contributed to a high false positive rate, indicating potential over-classification of risk levels by the sensor. Because the study did not include a low-risk group, it was not possible to identify false negatives, and it was not possible to calculate specificity. The 12-month predictive validity assessment for the Sensor yielded a sensitivity rate of 13.51% for true positives and a false positive rate of 86.48%. Additionally, the predictive sensitivity for identifying repeated falls within the high-risk group was 8%. To further evaluate the overall accuracy of the sensor in distinguishing between fallers and non-fallers, ROC analysis was conducted to provide a broader perspective on the sensor’s predictive capacity. The AUC value for the sensor was 0.688, indicating moderate accuracy in distinguishing between fallers and non-fallers.

## 4. Discussion

The findings of this study provide critical insights into the utility of the Sensor for classifying fall risk among community-dwelling older people, although significant limitations emerged regarding its predictive accuracy. It is disappointing to find that the Sensor is not useful for predicting prospective falls among older people over a prospective 12-month period because of its low sensitivity and high false positive rate, as well as the lack of significant differences in physical performance in the physical function tests between older people in the moderate or high-risk groups identified by the Sensor System. This aligns with the conclusion of a recent scoping review, which found that there was very limited evidence on the use of activity tracker-based wearables for the lower extremity for home-based rehabilitation in the stroke population [21]. One contributing factor might be the machine learning algorithm used for classifying fall risk, which could require refinement to achieve greater predictive accuracy. Although the sensor has been tested in laboratory environments, its effectiveness in real-world settings remains to be optimized.

To elaborate, the Sensor System’s limited predictive utility may stem from multiple sources, including the algorithm’s design and insufficient training data from participants who are fallers. This perspective is informed by a previous study reporting a higher sensitivity (30%) but a lower AUC (0.590) in a cross-sectional study analysis using the same device, indicating potential differences in performance based on study design [17]. In contrast, our prospective design yielded an AUC of 0.688, indicating moderate discriminatory ability, although sensitivity remained modest at 13.51%. This discrepancy highlights a key difference in that the prior study assessed concurrent fall risk, whereas our study evaluated predictive validity over 12 months, a more realistic fall measurement requiring robust extrapolation from baseline measurements. The high false positive rate (86.48%) further suggests that the algorithm may overestimate risk, potentially due to an overly conservative threshold or insufficient differentiation of subtle risk factors in community settings.

Another finding of this study is the discriminant validity of the sensor, which captures mainly the balance data due to body sway. Although balance is indeed a significant factor, our study showed only moderate accuracy in the SLST’s correct classification rate, suggesting that balance alone does not strongly correlate with fall risk levels. This reflects the multifactorial nature of fall risk, which involves not only balance but also other components such as muscle strength, reaction time, and postural control; multi-features constructed from various raw signals from sensors are essential for predictive models [30]. Research indicates that balance impairments elevate fall risk to a degree, but reliance on balance alone may overlook other critical risk factors [31]. For example, prior work found a relative risk of 1.42 for falls linked to balance impairment, a modest effect that emphasizes the importance of a comprehensive assessment [31]. Our SLST’s 51.4% classification accuracy supports this, indicating that while balance contributes to risk, its predictive capacity is constrained without integrating additional dimensions such as strength or cognitive performance.

In contrast, the higher classification accuracy of the 6MWT underscores the importance of walking speed as an indicator of functional mobility, which is essential for maintaining balance and fall prevention. Walking speed, as shown in other studies, is a critical factor in fall risk assessment, with slower gait speeds significantly linked to an increased risk of falls [32]. This finding highlights that screening for walking speed could be an effective measure for early fall risk detection. Additionally, the significant association highlighted by the 5STS’ classification rate suggests that lower body strength is a key component of fall risk, pointing to the importance of strengthening lower body capabilities to prevent falls. Strengthening interventions targeting lower body capabilities have been shown to reduce fall rates in older populations, aligning with our findings [33]. Specifically, the 6MWT’s 64.9% accuracy and the 5STS’s 59.5% accuracy suggest that mobility and strength metrics align more closely with fall risk than balance alone. Research has reported odds ratios of 2.84–4.88 for falls associated with slower gait speeds, reinforcing its significance [34]. Similarly, a systematic review concluded that lower limb muscle strengthening effectively reduces fall rates among older people, corroborating our 5STS results and reinforcing the relevance of such interventions in fall prevention [33]. These findings suggest that while the Sensor System captures these parameters, its algorithm may not fully leverage their predictive potential.

Among the parameters of fall risk, walking speed, measured through the 6MWT, prominently emerged as a pivotal metric, shown by the highest correct classification rate of fall risk. This result aligns with findings from multiple studies, which have shown diminished gait velocity in individuals with a history of falls [35,36]. In another recent study using decision tree analysis for a large sample of older people living in community dwellings in Hong Kong, a significant increase in fall risk was demonstrated among individuals with a slower score in the TUG, further supporting walking speed as a marker for mobility and fall risk [37]. The TUG test evaluates multiple aspects of physical function, including balance, gait, and lower limb strength, offering a more comprehensive assessment compared to walking speed alone [34]. There are international practice guidelines that highlight the TUG as the gold standard for fall risk assessment [38,39].

Despite the lack of statistically significant differences in physical function tests across both groups, the medium effect size observed in Group 2’s 6MWT suggests meaningful distinctions in mobility between risk groups. These results suggest that cognitive factors may be a contributing factor for slow walking speed, supporting previous research that highlights the critical interplay between cognitive function and physical mobility in determining fall risk [31]. In Group 2, where MoCA data were available, the 50% classification accuracy hints at a potential cognitive influence, though logistic regression analysis revealed no statistically significant association between HK-MoCA scores and fall occurrence over a prospective one-year period. Studies have reported that gait speed declines over 12 months increased fall risk in older people with mild cognitive impairment, suggesting a linkage that our sensor may not fully capture [30]. The absence of a strong association in our study is likely due to the small subset (n = 22), which limits statistical power. Additionally, with few people showing potential cognitive impairment, the sample may not reflect the impact of more severe cognitive decline on falls.

Falls are multifactorial, and even though the algorithm of the sensor measures three key parameters (balance, gait, and speed), the prediction of falls was still not accurate. Our study revealed the sensor’s tendency to overestimate fall risk, as evidenced by its low sensitivity rate of 13.51% true positives for fallers, and a high false positive rate of 86.48%. Additionally, ROC analysis provided an AUC value of 0.688, suggesting moderate performance in distinguishing between fallers and non-fallers. These findings are consistent with a recent study, which noted that despite capturing the aforementioned critical parameters for falls, the Sensor System still exhibited limitations in predictive accuracy for fall risk [17]. These results point to the need for further refinement in the sensor’s machine learning algorithms and for a broader range of predictors to enhance fall risk classification by the manufacturer.

Despite evidence in the literature affirming the potential of wearable technologies for fall risk assessment, the Sensor System exhibited limited predictive accuracy, likely due to its inability to incorporate contextual factors such as environmental hazards or participant comorbidities, in contrast to traditional tools like the TUG. Research indicates that while wearables excel in movement monitoring, their sensitivity for multifactorial prediction often requires extensive data inputs [23]. A prior investigation using the same device reported a sensitivity of 30% and an AUC of 0.590 in a cross-sectional design, whereas our prospective study, conducted over 12 months, yielded a lower sensitivity of 13.51% but a higher AUC of 0.688 [17]. This discrepancy underscores the distinct challenges inherent in predicting falls over an extended period compared to assessing immediate risk at a single time point. The reduced sensitivity of the Sensor in our study may reflect the complexity of forecasting prospective fall events based on baseline measurements alone, while the improved AUC suggests a moderate ability to differentiate fallers from non-fallers over time, highlighting a trade-off between immediate risk identification and predictive capacity of actual falls prospectively. This observation suggests that the current algorithm may be less suited for long-term fall prediction in real-world settings, which is time-consuming in study, and that dynamic changes in fall risk factors of an individual occur over time, potentially limiting its clinical utility for proactive interventions. Consequently, enhancing predictive accuracy may require longitudinal data collection beyond a single baseline assessment and the integration of additional variables, such as cognitive status or environmental conditions, to better capture the evolving nature of fall risk.

Wearable technologies, including the Sensor System, possess inherent advantages that could address these limitations, notably their capacity for continuous monitoring and objective data collection in daily life, surpassing the capabilities of clinical assessments such as the TUG, which demand trained personnel and controlled environments [15]. Such features enable the potential detection of transient risk events unobservable in traditional settings. Nevertheless, our findings indicate that these strengths have not yet fully translated into reliable fall prediction within the context of this study. The observed limitations may stem from the algorithm’s reliance on a 104-feature model that emphasizes laboratory-derived metrics, such as postural sway, over real-world variables, including fatigue or medication effects. Although raw data were unavailable to quantify the contributions of these factors, prior research supports the adaptation of algorithms to diverse populations, such as frail versus active elderly, as a viable approach to improvement [17].

Future advancements could involve retraining the algorithm with larger, more diverse datasets that include prospective fall incidents, alongside the incorporation of supplementary predictors like cognitive scores, comorbidity indices, or environmental data, to bolster predictive accuracy. Additionally, the study protocol, which relied on standardized tasks conducted at a single time point, may fail to account for dynamic changes in fall risk over the 12-month period, unlike longitudinal studies that employ repeated assessments [36]. In contrast, manufacturer trials, presumably conducted with larger cohorts under controlled conditions, differ markedly from the real-world constraints encountered in this community-based investigation. This disparity highlights the broader challenge of translating laboratory-validated tools into practical community applications.

## 5. Limitations

This study has several limitations. First, the small sample size limits the generalizability of our findings. With a biased sample of 37 participants, the sample might not be representative enough, potentially obscuring subtle associations between the sensor-derived fall risk predictions and actual fall events. In contrast to manufacturer trials, which presumably involved larger cohorts under controlled conditions with unspecified participant numbers due to proprietary restrictions, this modest sample reflects real-world feasibility rather than an optimal research design. Although constrained by resource availability, the limited size restricts the applicability of conclusions to broader elderly demographics, necessitating future studies with expanded, more representative cohorts to enhance the robustness and external validity of the sensor’s performance evaluation.

Second, the study excluded low-risk and no-risk fall groups to prioritize service resources for those at higher risk. This pragmatic decision, driven by the need to allocate limited clinical support to individuals identified as moderate or high risk by the sensor, introduced a selection bias that narrowed the assessment scope. Consequently, the ability to evaluate the sensor’s performance across the full spectrum of fall risk, including its capacity to correctly classify non-fallers and minimize false negatives, was compromised. While this focus ensured targeted resource use, it may have contributed to the elevated false positive rate of 86.48%. In contrast, studies incorporating all risk levels could offer a more comprehensive validation of discriminant capabilities, suggesting a need for future research to address this methodological trade-off by including a wider range of risk categories.

Third, cognitive function data, measured by the MoCA, were available for only a subset of participants due to resource constraints, restricting analysis of cognitive factors in fall risk prediction. Administering the HK-MoCA to only 22 of 37 participants resulted from time and staffing limitations, precluding a full cohort assessment. Logistic regression analysis revealed no statistically significant association between HK-MoCA scores and fall occurrence over the one-year period, likely influenced by the reduced sample size and the predominance of participants scoring above the cutoff, with 19 of 22 showing no severe cognitive deficits. This incomplete dataset limited the exploration of cognitive influences on falls, necessitating cautious interpretation and highlighting the value of future studies with comprehensive cognitive data collection to clarify this relationship.

Fourth, the study did not include information on comorbidities and medications, which are potential covariates in fall risk analysis. Data collection emphasized sensor-derived metrics, such as balance, gait, and speed, omitting health-related factors like diabetes, hypertension, or polypharmacy due to logistical difficulties in obtaining medical records from community participants and the study’s defined scope. These unmeasured variables, prevalent among older people, likely introduced variability unaccounted for by the algorithm. Medications, particularly those affecting neurological or cardiovascular function, such as sedatives or beta-blockers, could alter mobility and fall risk independently of sensor parameters. This gap represents a critical limitation, as the absence of such covariates may reduce the predictive model’s granularity, hindering accurate application in community settings. Future research should integrate these factors to bridge the divide between controlled and naturalistic environments.

Finally, we were unable to conduct a linear regression analysis using the raw data from the sensor from the manufacturer. The proprietary algorithm’s design prevented access to detailed kinematic outputs, such as raw accelerometer or gyroscope readings, a significant barrier to quantifying feature contributions within the predictive model or assessing their relative impact on fall risk classification. Manufacturer trials, presumably conducted with larger cohorts and unrestricted data under controlled conditions with undisclosed participant numbers, differ markedly from the real-world constraints of this community-based study. Without raw data, we could not examine why the sensor overestimated risk, as indicated by the false positive rate of 86.48%, or refine the model independently, despite prior recommendations to tailor algorithms to specific populations [17]. This limitation reflects a broader issue, as the lack of transparency in commercial wearable systems hampers independent validation and optimization, underscoring the need for future collaboration with manufacturers or the development of open-source alternatives to enable detailed statistical analyses and enhance real-world efficacy.

## 6. Conclusions

This study investigated the sensitivity of a waist-belt wearable Sensor System in evaluating and predicting fall risk among older people living in community dwellings over a prospective 12-month period. The investigation revealed that the Sensor System demonstrated limited efficacy in forecasting actual fall occurrences, attributable to its low sensitivity, which accurately identified only a minimal proportion of fallers, and a substantially elevated false positive rate, frequently misclassifying individuals not at risk as susceptible. Moreover, the Sensor’s risk classifications exhibited inconsistent correspondence with established physical function assessments, encompassing measures of balance, mobility, and lower limb strength, across participants designated as moderate or high risk. These outcomes collectively indicate that the waist-mounted sensor, in its current iteration, does not constitute a reliable instrument for predicting fall incidents among older people in community settings, diverging from the anticipated capabilities of wearable technologies for fall risk assessment. Future research should prioritize the implementation of longitudinal monitoring protocols, extending beyond a singular baseline assessment, and the incorporation of a broader spectrum of factors, including chronic morbidities, medications affecting neurological or cardiovascular function, and environmental variables reflective of dynamic risk profiles in community contexts. Validation and sensitivity studies of various sensors may involve collaborative efforts with technology developers to access proprietary data or the pursuit of transparent, adaptable systems, ensuring that wearable sensors realize their potential as effective instruments for mitigating prospective fall risks and promoting the well-being of older people in community dwellings.

## Figures and Tables

**Table 1:** Comparison of demographics and baselines in Model 1 and Model 2.

Characteristics	Group 1			Group 2		
	High-Risk Group (n = 25)	Moderate-Risk Group (n = 12)	*p*	High-Risk Group (n = 16)	Moderate-Risk Group (n = 6)	*p*
Age (year), mean ± SD	71.56 ± 5.01	72.33 ± 3.80	0.64	71.63 ± 5.15	72.33 ± 4.23	0.77
Gender (%)						
Male	6 (24)	2 (16.7)		5 (31.25)	0 (0)	
Female	19 (76)	10 (83.33)		11 (68.75)	6 (100)	
No. of falls in past 12 months (%)	4 (16)	2 (16.67)		3 (18.75)	0 (0)	
On >4 medications (%)	4 (12)	2 (16.7)		3 (18.75)	1 (16.7)	

**Table 2:** Classification of fall risk level by wearable sensor using SLST, 6MWT, and 5STS.

Fall Risk Level	^1^ Predicted Group Membership, n (%), Using SLST		
	Moderate	High	Total
Moderate	6 (50)	6 (50)	12
High	12 (48)	13 (52)	25
	^2^ Predicted Group Membership, n (%), using 6MWT		
	Moderate	High	Total
Moderate	7 (58.3)	5 (41.7)	12
High	8 (32)	17 (68)	25
	^3^ Predicted Group Membership, n (%), using 5STS		
	Moderate	High	Total
Moderate	4 (33.3)	8 (66.7)	12
High	7 (28)	18 (72)	25

SLST = Single Leg Stand Test; 6MWT = 6 Minute Walk Test; 5STS = 5 Sit to Stand Test; ^1^ 51.4% of original grouped cases correctly classified; ^2^ 64.9 % of original grouped cases correctly classified; ^3^ 59.5 % of original grouped cases correctly classified.

**Table 3:** Classification of fall risk level by wearable sensor using HK-MoCA.

Fall Risk Level	^1^ Predicted Group Membership, n (%), Using MoCA		
	Moderate	High	Total
Moderate	4 (66.7)	2 (33.3)	6
High	9 (56.3)	7 (43.8)	16

MoCA = Montreal Cognitive Assessment; ^1^ 50.0% of original grouped cases correctly classified.

**Table 4:** Predictive validity confusion matrix for the wearable sensor.

Risk Classification by the Sensor	Actual Outcomes	
	Actual Fall (%)	No Fall (%)
High-risk group	5 (13.51)	20 (54.05)
Moderate-risk group	0 (0)	12 (32.43)

## Data Availability

The data and algorithms presented in this study are available from the corresponding author upon reasonable request.
